# Prevalence of Germline *BRCA1/2* Variants in Ashkenazi and Non-Ashkenazi Prostate Cancer Populations: A Systematic Review and Meta-Analysis

**DOI:** 10.3390/cancers15010306

**Published:** 2023-01-02

**Authors:** Antonio Cioffi, Ottavio De Cobelli, Paolo Veronesi, Carlo La Vecchia, Patrick Maisonneuve, Giovanni Corso

**Affiliations:** 1 Division of Urology, European Institute of Oncology (IEO), IRCCS, 20141 Milan, Italy; 2 Department of Oncology and Hemato-Oncology, University of Milan, 20122 Milan, Italy; 3 Division of Breast Surgery, European Institute of Oncology (IEO), IRCCS, 20141 Milan, Italy; 4 Department of Clinical Sciences and Community Health, University of Milan, 20122 Milan, Italy; 5 Division of Epidemiology and Biostatistics, European Institute of Oncology (IEO), IRCCS, 20141 Milan, Italy; 6 European Cancer Prevention Organization (ECP), 20122 Milan, Italy

**Keywords:** prostate cancer, *BRCA1* mutations, *BRCA2* mutations, Ashkenazi, meta-analysis

## Abstract

**Simple Summary:**

Germline *BRCA2* pathogenic variant carriers are associated with prostate cancer risk. Ashkenazi Jewish people are at higher risk of breast cancer due to the high prevalence of specific founder germline *BRCA1/2* variants. The distribution of these variants (*BRCA1* vs. *BRCA2*) in Ashkenazi men with prostate cancer is not clear. This systematic review and meta-analysis indicates that germline *BRCA1* variants are higher in the Ashkenazi Jewish ethnicity in comparison to non-Ashkenazi men. Instead, *BRCA2* variants present a similar distribution between the two considered groups.

**Abstract:**

Background and aims: International guidelines recommend testing *BRCA2* in men with prostate cancer, due to the presence of a strong association with this gene. Some ethnicities present disparities in genetic distribution for the relation with specific founder variants. Ashkenazi Jewish people are, importantly, at high risk of breast cancer for their inherited cluster with germline *BRCA1/2* variants. However, in Ashkenazi men with prostate cancer, the prevalence of *BRCA1* and/or *BRCA2* is not well defined. We assessed the frequency of these variants in Ashkenazi vs. non-Ashkenazi men with prostate cancer. *Materials and Methods*: In accord with the Preferred Reporting Items for Systematic reviews and Meta-Analyses (PRISMA) statement, we revised all germline *BRCA* variants reported in MEDLINE from 1996 to 2021 in Ashkenazi and non-Ashkenazi men with prostate cancer. *Results:* Thirty-five original studies were selected for the analysis. Among populations from Israel and North America, Ashkenazi Jewish men presented higher prevalence of *BRCA1* variants [0.9% (0.4–1.5) vs. 0.5% (0.2–1.1), *p* = 0.09] and a lower prevalence of *BRCA2* variants [1.5% (1.1–2.0) vs. 3.5% (1.7–5.9), *p* = 0.08] in comparison to the non-Ashkenazi population. *Conclusions:* Since germline *BRCA1* variants are more prevalent and *BRCA2* variants are less prevalent in PCa patients of Ashkenazi Jewish ethnicity in comparison to non-Ashkenazi patients, prostate cancer genetic screening in Ashkenazi men should not be restricted to the *BRCA2* gene.

## 1. Introduction

Prostate cancer (PCa) is the second most commonly diagnosed cancer in men, accounting for 15% of all male cancers [1]. Family history and ethnic background are associated with an increased incidence, even if defined hereditary disease is present in only about 10% of men with PCa [2,3].

Many factors have been associated with the risk of PCa or as being important for disease progression [4]. Obesity has been associated with an increased risk of high-grade PCa [5], and high alcohol intake has been associated with a higher risk of PCa and PCa-specific mortality [6]. An organized program of PCa screening may contribute to a further 5–10% reduction in cancer mortality [7,8].

Inherited PCa predisposition accounts in about 10% of families [9]. Genetic screening demonstrated a strong association with germline *BRCA2* mutations and PCa risk. However, there are no standardized measures for risk containing in asymptomatic high-risk individuals [10].

Ashkenazi Jewish people have high breast cancer (BC) risk due to the high penetrance of some specific germline *BRCA* variants in their ancestry, in particular c.185delA/ 5382insC for *BRCA1* and c.6174delT for *BRCA2* [11]. In BC Ashkenazi Jewish people with positive germline *BRCA1/2* variants, *BRCA1* is prevalent (71%) in comparison to *BRCA2* (29%) [12]. The global prevalence of *BRCA1/2* variants in Ashkenazi Jewish people with PCa is undefined. With the present study, we revised the prevalence of germline *BRCA1/2* variants in Ashkenazi and non-Ashkenazi populations using a systematic review and meta-analysis approach. 

## 2. Methods

### 2.1. Data Retrieval

**Figure 1:**
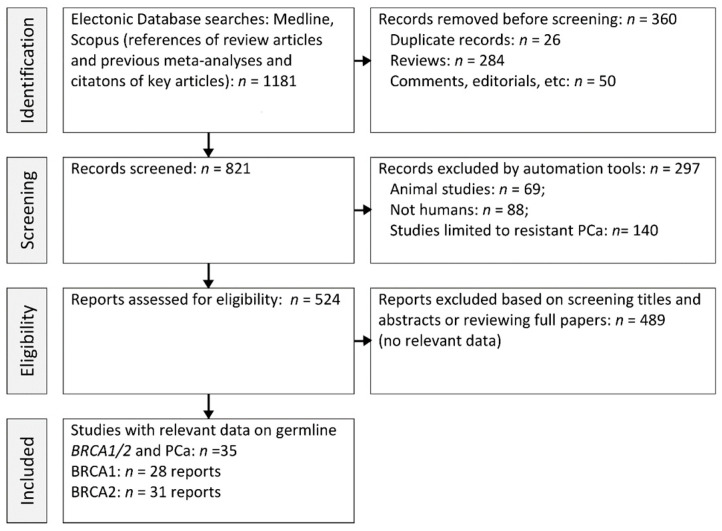
PRISMA flow diagram illustrating the literature search.

We used the checklist of items in accordance with the Preferred Reporting Items for Systematic reviews and Meta-Analyses (PRISMA) statement (Figure 1). We revised all germline *BRCA* variants reported in MEDLINE from 1996 to 2021, including original reports and literature reviews edited in English language. We used the following PubMed query for the initial search: “[Prostate cancer and (*BRCA* OR *BRCA1* OR *BRCA2*)] AND 1996/01:2021/12[EDAT]”. We also reviewed references and citations of key articles and previous meta-analyses on the association between *BRCA1/2* variants and PCa using Scopus. The analysis was limited to studies involving subjects affected by PCa screened for germline *BRCA* variants (Figure 1). This study was not registered.

### 2.2. Study Selection

Original studies that contained information on both germline *BRCA1/2* and PCa were considered. Case series, editorials, case reports, and congress abstracts were excluded. Titles and abstracts were screened by two authors to determine whether studies met the eligibility criteria for full-text assessment, and a sample of 25% was screened independently by another author as a quality assurance process. The full-text assessment was conducted by two authors with consultation from a third author if required. Full-text publications in English were considered. Case series, editorials, case reports, and congress abstracts were excluded from the evidence synthesis.

### 2.3. Data Extraction

Data extraction was performed by one author (PM), with another independent extraction by one of two other authors (GC and AC). Any disagreements were resolved by discussion and consensus. The following data were extracted using predefined cells: first author, publication date, country, screening interval, ethnicity, number of identified *BRCA1* and *BRCA2* variants, and number of tested PCa.

### 2.4. Data Analysis

For each study, we first estimated the prevalence of *BRCA1/2* variants in PCa from the published reports. We calculated 95% confidence intervals (CIs) using the Clopper–Pearson Exact binomial method. For the meta-analysis, we used random-effects models, with maximum likelihood estimates and the Freeman–Tukey double arcsine transform method to stabilize variances. Heterogeneity between studies was assessed using the Cochran Q test. The percentage of total variation across studies due to heterogeneity was evaluated by the I2 measure. We produced forest plots including the study specifics and the overall prevalence estimates. Probability of publication bias was assessed using funnel plots with the Begg’s test. Statistical analyses were performed using the R package meta. All *p*-values were two-sided.

## 3. Results

### 3.1. Study Features

We selected 35 original studies published from 1996 to 2021. These reported data were collected from 1983 to 2017. A total of 27,252 PCas were screened for *BRCA1* and 24,633 for *BRCA2*. Three studies comprised heterogeneous populations [13,14,15], and eleven studies included Ashkenazi PCa patients for *BRCA* screening (Table 1). 

### 3.2. Prevalence of BRCA1/2 Mutations

**Figure 2:**
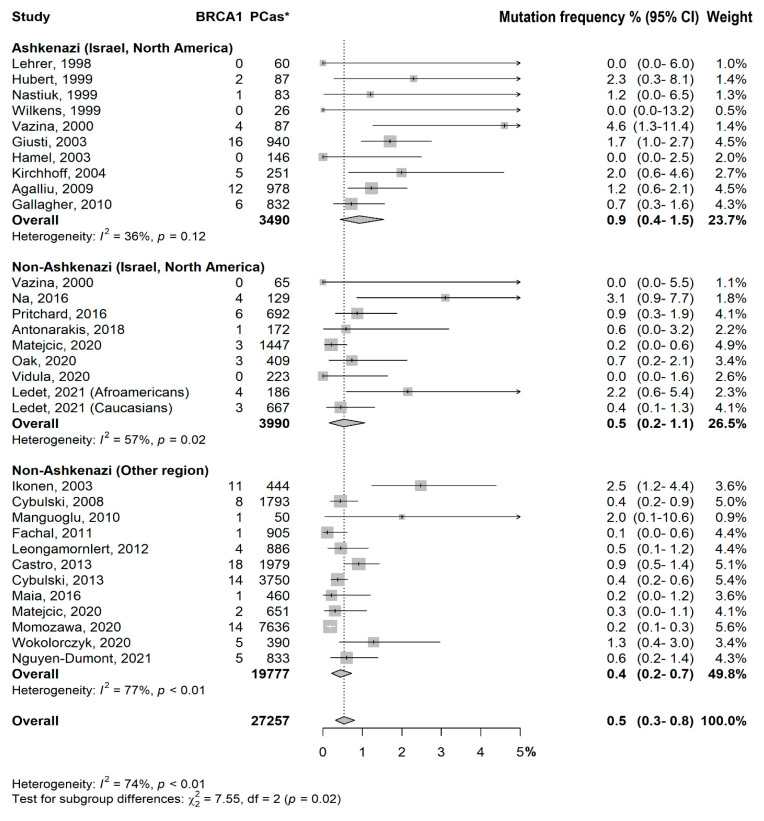
*BRCA1* forest plot distribution. Prevalence in percent of *BRCA1* variants among different populations (Ashkenazi vs. non-Ashkenazi) coming from different countries (Israel/north America vs. other regions). *PCas: Number of prostate cancer cases.

**Figure 3:**
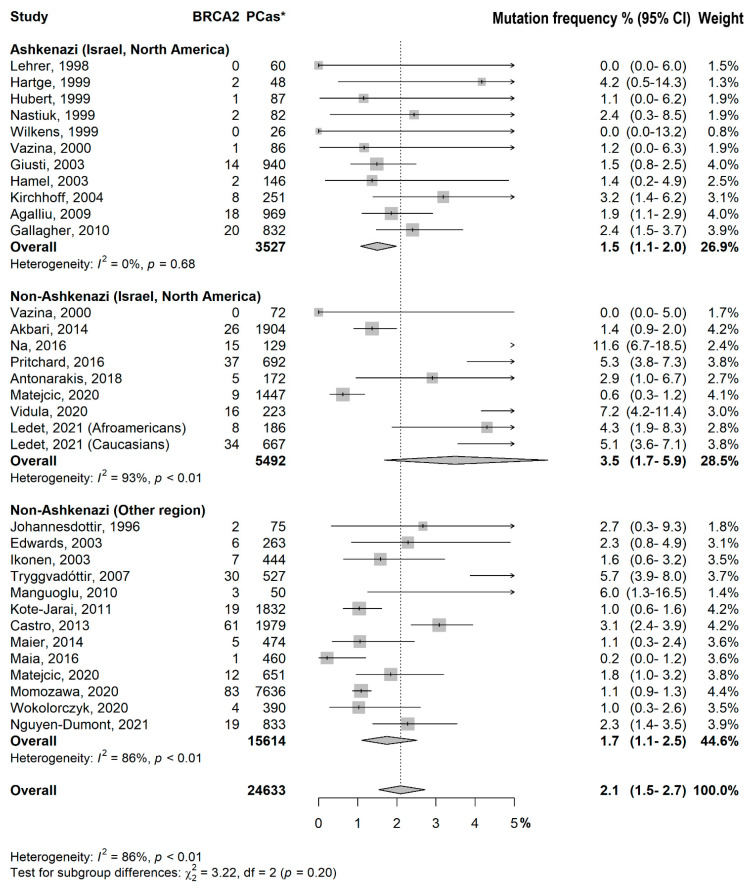
*BRCA2* forest plot distribution. Prevalence in percent of *BRCA2* variants among different populations (Ashkenazi vs. non-Ashkenazi) coming from different countries (Israel/north America vs. other regions). *PCas: Number of prostate cancer cases.

We identified 46 *BRCA1* and 68 *BRCA2* variants in PCa Ashkenazi patients, and 108 *BRCA1* and 402 *BRCA2* variants in non-Ashkenazi patients. Regarding the classic founder *BRCA1/2* variants in Ashkenazi patients, we identified 185delAG in thirty-nine, 5382insC in seven, and 6176delT in sixty-eight patients, respectively. Considering the overall PCa patients, in Ashkenazi patients we identified a total of 46 (40.4%) germline *BRCA1* (3,490 PCas) and 68 (59.6%) *BRCA2* (3,527 PCas) variants, and in non-Ashkenazi patients 108 (21.2%) *BRCA1* (23,767 PCas) and 402 (78.8%) *BRCA2* (21,106 PCas) variants. Forest plot analyses of variant frequency in percent are reported in Figure 2 for *BRCA1* and in Figure 3 for *BRCA2*. We observed that Ashkenazi Jewish patients coming from Israel and North America had higher prevalence of *BRCA1* variants in comparison to non-Ashkenazi populations from Israel and North America [0.9% (0.4–1.5) vs. 0.5% (0.2–1.1); *p* = 0.09], and also from other countries [0.4 (0.2–0.7); *p* < 0.006]. No significant difference was observed in the prevalence of *BRCA1* variants among non-Ashkenazi patients from Israel and North America, and non-Ashkenazi patients from other regions (*p* = 0.50) (Figure 2). 

Regarding *BRCA2* variant status, Ashkenazi (Israel and North America) patients had a lower prevalence of variants, in comparison to the similar non-Ashkenazi population [1.5% (1.1–2.0) vs. 3.5% (1.7–5.9); *p* = 0.08]. 

Studies from non-Ashkenazi populations had a significant heterogeneity in *BRCA1* and *BRCA2* groups (*p* < 0.01), both (Figure 1 and Figure 2).

In Supplementary Table S1, we described the pathogenic classification of the germline *BRCA1/2* variants reported in the respective original studies. When a variant’s nomenclature was available in the text, we revised the pathogenic role of these alterations according to the most recent *ClinVar* classification (Supplementary Table S2).

Test for subgroup differences:

− Ashkenazi (Israel, North America) vs. Non-Ashkenazi (Israel, North America): *p* = 0.09
− Ashkenazi (Israel, North America) vs. Non-Ashkenazi (Other region): *p* = 0.006
− Non-Ashkenazi (Israel, North America) vs. Non-Ashkenazi (Other region): *p* = 0.50
− Test for subgroup differences:
− Ashkenazi (Israel, North America) vs. Non-Ashkenazi (Israel, North America): *p* = 0.08
− Ashkenazi (Israel, North America) vs. Non-Ashkenazi (Other region): *p* = 0.86
− Non-Ashkenazi (Israel, North America) vs. Non-Ashkenazi (Other region): *p* = 0.08

### 3.3. Risk of Bias

**Figure 4:**
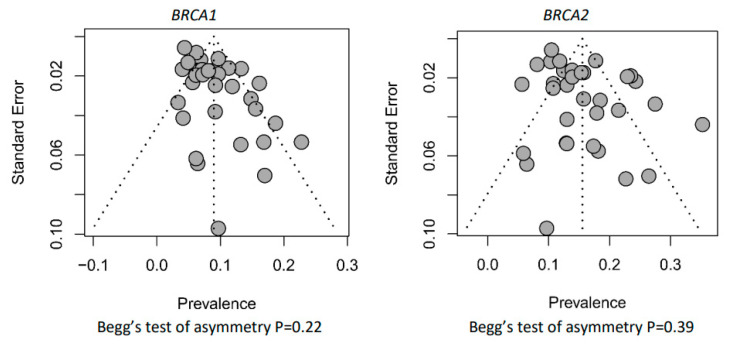
*BRCA1/2* funnel plot distribution.

Examination of the funnel plot revealed no evidence of publication bias (*p* = 0.22 and *p* = 0.39, respectively, for *BRCA1* and *BRCA2* by Begg’s test) (Figure 4).

## 4. Discussion

### 4.1. Hereditary PCa in Ashkenazi Is Associated with Germline BRCA Mutation Status

This study presents a different distribution of germline *BRCA1/2* variants among PCa Ashkenazi and non-Ashkenazi patients. We identified a higher prevalence of germline *BRCA1* variants in Ashkenazi Jewish patients in comparison to the non-Ashkenazi population, while no significant difference of the prevalence of germline *BRCA2* variants was observed between the two populations. 

Some ethnicities present a specific relation with founder germline gene variants. Significant associations were found in Māori kindred carrying *CDH1* germline pathogenic variants for gastric cancer risk, as well as in Ashkenazy Jewish and Hakka Chinese populations with *BRCA* variants for BC risk [48].

With reference to the *BRCA* gene and Ashkenazi patients, there is a documented relation with BC risk. The 185delAG and 5382insC variants in *BRCA1* and the 6174delT variant in *BRCA2* were detected in a high proportion of Ashkenazi Jewish women with a diagnosis of BC. Initially, the 185delAG variant was found in 17%, 5382insC in 2%, and 6174delT in 6% of cases [49]. These three founder variants account for the vast majority (~96%) of *BRCA1/2* variants in women of Ashkenazi Jewish descent [50]. Specifically, a national Israeli study of 111 Ashkenazi families showed that among families in which the *BRCA1* 185delAG variant was detected, 20.2% of the women had BC. A similar incidence of 24.1% was found among women from families with the *BRCA2* 6174delT variant. Among families carrying the *BRCA1* 5382insC variant, the incidence was appreciated higher (39.4%) [12]. We noted a different prevalence of founder variants in PCa Ashkenazi patients. In our study we identified a total of 114 founder germline *BRCA1/2* variants in Ashkenazi patients. In detail, the most frequent variants were 6174delT (59.7%), 185delAG (34.2%), and 5382insC (6.1%), respectively. In comparison to BC, the founder 6174delT (*BRCA2*) and 185delAG (*BRCA1*) variants were more prevalent in PCa. 

### 4.2. BRCA2 Is Confirmed the Hallmark of Pca in Non-Ashkenazi Populations

Disparity in Pca incidence and mortality across different countries and geographical areas might, at least in part, be related to differences in race, ethnicity, and genetic causes [51]. About 10% of PCa accounts as a hereditary form [9]. The main characteristic of this hereditary form is the early-onset manifestation. There are several genes associated with hereditary PCa, with variable frequencies: *HOXB13* (0.6–6.25%), *BRCA2* (1.2–5.3%), *CHEK2* (1.6–2.7%), *ATM* (1.6–2.7%), *MMR* (0.7–1.74%), *BRCA1* (0.9–1.25%), *PALB2* (0.4- 0.5%), *BRP1*, and *NBS1* (0.1–0.2%) [51,52,53]. Additionally, a rare inherited predisposition was also documented with germline *CDH1* variants, described in some families with hereditary PCa phenotype [54]. A study by Nyberg et al. [55] estimated PCa risk in a large population of *BRCA1* and *BRCA2* variant carriers. They identified 16 PCas in 376 *BRCA1* (4.2%) and 26 PCas in 447 *BRCA2* (5.8%) variant carriers during a median follow-up of about 5 years. They estimated a cumulative risk of developing PCa by age 85 years of 29% (95% CI 17–45%) for *BRCA1* and of 60% (95% CI 43–78%) for *BRCA2* carriers. Positive family history was associated with a stronger association in *BRCA2* carriers [7.31 (95% CI 3.40–15.72)]. In general, *BRCA2* carriers are at a two to five times higher risk of PCa compared to men in the general population. In contrast, *BRCA1* variants are at most associated with a moderate PCa risk [55]. In our study, we confirmed that germline *BRCA2* mutations are prevalent in non-Ashkenazi populations. 

### 4.3. Further Indications for PC Genetic Screening

The National Comprehensive Cancer Network (NCCN) recommends a *BRCA2* genetic test starting at age 40 years in cases of a personal history of metastatic, node-positive, biochemically recurrent, Gleason ≥8, or Gleason 7 disease with a strong family history of *BRCA*-related cancers. Additional men with PCa were referred if their personal and/or family history was suspicious for hereditary cancer predisposition, such as Lynch Syndrome [56]. However, *BRCA1* is not routinely included in PCa genetic screening but only “considered”, due to the lower prevalence in men with PCa. Our results demonstrate a similar prevalence of *BRCA1* and *BRCA2* in PCa Ashkenazi Jewish patients. This suggests “recommending” both genes in their genetic testing to inform decision-making for active surveillance. 

### 4.4. Limitations

The germline *BRCA1/2* variant frequency could be affected by research activity, publication bias or access to medical care, and some data could be not retrievable from MEDLINE. However, we assume that this potential bias is very low. Data collection was performed independently by two authors. 

We were not able to quantify the real PCa risk in *BRCA1* and *BRCA2* due to the lack of information about healthy carriers. However, our first aim was to assess only the prevalence of *BRCA* variants between two different populations. 

Some studies reported in this systematic review performed genetic screening in Ashkenazi Jewish patients only considering the *BRCA1/2* “hotspot” points such as 185delAG, 5382insC, 6174delT, and not all the entire coding regions. We can consider this approach as a “safe procedure” because founder variants are classically identified at a higher (significant) frequency in these populations. 

We collected all germline *BRCA* variants reported in these studies. Some of these studies did not specify if these variants are variants of unknown significance (VUS) or pathogenetic [22,25,28,37,39,40,41,46], as they reported only the overall number of identified mutations; other studies [31,34,38] included both VUS and pathogenetic germline *BRCA* variants. To provide additional information for further studies, when variants’ nomenclature was available in the original study, we have revised these alterations in accord with the most recent *ClinVar* classification.

## 5. Conclusions

Ashkenazi Jewish ancestry was associated with *BRCA1/2* variants in men with PCa, with a prevalence of *BRCA2* (1–3%) [20,26,30]. This meta-analysis suggests that *BRCA1* and *BRCA2* occur in PCa Ashkenazi with a similar prevalence; moreover, the prevalence of germline founder variants presents a different frequency in PCa in comparison to BC, in particular with 6176delT (*BRCA2*) and 185delAG (*BRCA1*) variants. This should be considered for active surveillance in Ashkenazi men. 

## Figures and Tables

**Table 1:** List of selected original studies reported in literature with information about BRCA variant status in Ashkenazi and non-Ashkenazi men with prostate cancer.

Study	Country	Period	Ethnicity	BRCA1 *	Tested **	BRCA2 *	Tested **
Johannesdottir, 1996 [16]	Iceland	1983-1992	Non-Ashkenazi	-	-	2	75
Lehrer, 1998 [17]	USA	-	Ashkenazi	0	60	0	60
Hartge, 1999 [18]	USA	1996	Ashkenazi	-	-	2	48
Hubert, 1999 [19]	Israel	-	Ashkenazi	2	87	1	87
Nastiuk, 1999 [20]	USA	1991–1996	Ashkenazi	1	83	2	82
Wilkens, 1999 [21]	USA	-	Ashkenazi	0	26	0	26
Vazina, 2000 [13]	Israel	1998	Ashkenazi	4	87	1	86
Vazina, 2000 [13]	Israel	1998	Non-Ashkenazi	0	65	0	72
Edwards, 2003 [22]	UK	1992–1999	Non-Ashkenazi	-	-	6	263
Giusti, 2003 [23]	Israel	1994–1995	Ashkenazi	16	940	14	940
Hamel, 2003 [24]	Canada	1991–2002	Ashkenazi	0	146	2	146
Ikonen, 2003 [25]	Finland	1996–1999	Non-Ashkenazi	11	444	7	444
Kirchhoff, 2004 [26]	USA	2000–2002	Ashkenazi	5	251	8	251
Tryggvadóttir, 2007 [27]	Iceland	1955–2004	Non-Ashkenazi	-	-	30	527
Cybulski, 2008 [28]	Poland	1999–2005	Non-Ashkenazi	8	1793		-
Agalliu, 2009 [29]	USA	1998–2005	Ashkenazi	12	978	18	969
Gallagher, 2010 [30]	USA	1988–2007	Ashkenazi	6	832	20	832
Manguoglu, 2010 [31]	Turkey	-	Non-Ashkenazi	1	50	3	50
Fachal, 2011 [32]	Spain	2006–2009	Non-Ashkenazi	1	905	-	-
Kote-Jarai, 2011 [33]	UK	-	Non-Ashkenazi	-	-	19	1832
Leongamornlert, 2012 [34]	UK	-	Non-Ashkenazi	4	886	-	-
Castro, 2013 [35]	Spain	-	Non-Ashkenazi	18	1979	61	1979
Cybulski, 2013 [36]	Poland	1999–2012	Non-Ashkenazi	14	3750	-	-
Akbari, 2014 [37]	Canada	1998–2010	Non-Ashkenazi	-	-	26	1904
Maier, 2014 [38]	Germany	1998–2007	Non-Ashkenazi	-	-	5	474
Maia, 2016 [39]	Portugal	-	Non-Ashkenazi	1	460	1	460
Na, 2016 [40]	USA	-	Non-Ashkenazi	4	129	15	129
Pritchard, 2016 [41]	USA	-	Non-Ashkenazi	6	692	37	692
Antonarakis, 2018 [42]	USA	-	Non-Ashkenazi	1	172	5	172
Matejcic, 2020 [14]	USA	1993–1996	Non-Ashkenazi	3	1447	9	1447
Matejcic, 2020 [14]	Uganda	1993–1996	Non-Ashkenazi	2	651	12	651
Momozawa, 2020 [43]	Japan	-	Non-Ashkenazi	14	7636	83	7636
Oak, 2020 [44]	USA	-	Non-Ashkenazi	3	409	-	-
Vidula, 2020 [45]	USA	2016–2017	Non-Ashkenazi	0	223	16	223
Wokolorczyk, 2020 [46]	Poland	2000–2017	Non-Ashkenazi	5	390	4	390
Ledet, 2021 [15]	USA, black	-	Non-Ashkenazi	4	186	8	186
Ledet, 2021 [15]	USA, white	-	Non-Ashkenazi	3	667	34	667
Nguyen-Dumon, 2021 [47]	Australia	-	Non-Ashkenazi	5	833	19	833

* Number of identified variants in BRCA1/2 genes. ** Overall number of screened patients with prostate cancer.

## Supplementary Materials

The following supporting information can be downloaded at: https://www.mdpi.com/article/10.3390/cancers15010306/s1, Table S1: Germline BRCA1/2 pathogenic classification reported in the original studies. Table S2: Pathogenicity of BRCA1/2 variants.
