# Supplementary material for: Prevalence of Germline BRCA1/2 Variants in Ashkenazi and Non-Ashkenazi Prostate Cancer Populations: A Systematic Review and Meta-Analysis
Source: Cancers (Basel). 2023 Jan 2;15(1):306. doi: 10.3390/cancers15010306 (PMC9818251; doi:10.3390/cancers15010306)
Supplement: Supplementary file 1 [file cancers-15-00306-s001.zip › cancers-2084904-supplementary.pdf]

Table S1. Germline *BRCA1/2* pathogenic classification reported in the original studies.

Study	*BRCA1/2*	P/LP	VUS	Unknown
Johannesdottir, 1996 [16]	2	-	2	-
Hartge, 1999 [18]	2	2	-	-
Hubert, 1999 [19]	3	3	-	-
Nastiuk, 1999 [20]	3	3	-	-
Vazina, 2000 [13]	5	5	-	-
Edwards, 2003 [22]	6	-	-	6
Giusti, 2003 [23]	30	30	-	-
Hamel, 2003 [24]	2	2	-	-
Ikonen, 2003 [25]	18	-	-	18
Kirchhoff, 2004 [26]	13	13	-	-
Tryggvadóttir, 2007 [27]	30	30	-	-
Cybulski, 2008 [28]	8	-	-	8
Agalliu, 2009 [29]	30	30	-	-
Gallagher, 2010 [30]	26	26	-	-
Manguoglu, 2010 [31]	4	-	4	-
Fachal, 2011 [32]	1	P		-
Kote-Jarai, 2011 [33]	19	19	-	-
Leongamornlert, 2012 [34]	4	4	-	-
Castro, 2013 [35]	79	-	-	79
Cybulski, 2013 [36]	14	14	-	-
Akbari, 2014 [37]	26	-	-	26
Maier, 2014 [38]	5	5	-	-
Maia, 2016 [39]	2	-	-	2
Na, 2016 [40]	19	-	-	19
Pritchard, 2016 [41]	43	-	-	43
Antonarakis, 2018 [42]	6	6	-	-
Matejcic, 2020 [14]	12	12	-	-
Matejcic, 2020 [14]	14	14	-	-
Momozawa, 2020 [43]	97	97	-	-
Oak, 2020 [44]	3	-	-	3
Vidula, 2020 [45]	16	16	-	-
Wokolorczyk, 2020 [46]	9	-	-	9
Ledet, 2021 [15]	12	12	-	-
Ledet, 2021 [15]	37	37	-	-
Nguyen-Dumon, 2021 [47]	24	24	-	-

P/LP: Pathogenic/ Likely Pathogenic; VUS: Variant of Unknown Significance. Unknown: The pathogenicity was not defined in the study.

Table S2. Pathogenicity of BRCA1/2 variants.

HGVS (coding region)	*ClinVar* classification
999del5*	P
6174delT**	P
185delAG**	P
5382insC**	P
1231delA	P
1265delA	P
1787delATGAAACATCTTA	NS
1813insA	P
2807delAACA	VUS
2836delGA	P
3158T>G	VUS
3405C>A	P

3847delGT	P
4478delAAAG	NS
4877delAA	NS
4981delT	P
5303delTT	NS
5645C>A	P
6405delCTTAA	P
8904delC	P
9253insA	P
211A>G	P
68_69delAG	P
212+1G>T	P
1954dupA	P
2475delC	P
181T>G***	P
1813_14_insA	P
3847delGT	P
4449delA	P
6037A>T	P
7495C>T	NS
4956G>A	B
5744C>T	B
7469T>C	B
8482A>G	VUS
3893CA	NS
5946delT	P
9076T>C	NS
5946delT	P
349A>G	P/LP
9285C>T	LB
1049delA	P
1204delG	P
1800T>A	P
1970T>A	P
2806delA	P
3719_3722del	P
3862_3865del	P
4127_4130del	P
4708_4709del	P
5473_5474insCAGC	P
5632dupA	P
5769_5772del	P
5946delT	P
6124C>T	P
7024C>T	P
7203_7206del	P
8755-2A>G	P
8897_8898insCACAA	P
8970G>A	P
9247dupA	P
9382C>T	P
9692C>A	P
9890_9891insATTT	P
316+2T>C	P
632-1G>A	P
893_894dupCA	NS

1184G>A	P
1261C>T	P
1399A>T	P
1813delA	P
1825C>T	P
2548_2549insCC	NS
2670delT	NS
3599_3600delGT	P
3649delA	P
3744_3747delTGAG	P
3940A>T	NS
4285dupC	NS
4339delG	P
4464_4465delCA	NS
4649_4650delAG	NS
4822G>T	NS
4952delC	P
5081_5084delGAGA	NS
5482_5486delAAATT	P
5576_5579delTTAA	P
5598dupT	NS
5635G>T	P
5645C>A	P
5709delT	NS
5722_5723delCT	P
6063_6066delTTCA	NS
6405_6409delCTTAA	P
6408_6414delAAATGTT	P
6446_6450delTTAAA	P
6468_6469delTC	P/LP
6462T>G	P
6553delG	P
6938-2A>G	P
6952C>T	P
7480C>T	P
7615C>T	P
7976+1G>T	NS
8023A>G	P/LP
8168A>G	P
8504C>A	P
8589dupA	NS
8629G>T	P
9053delG	NS
9076C>T	P
9097dupA	NS
9155G>A	P
10061delC	NS
10062delT	NS
10220_10223delATAA	NS
2899A>T	NS
2834+2T>C	NS
2480_2481delCA	NS
1671_1674delTATT	P
514_517delTCTG	NS
49-1delG	NS
5575G>T	VUS

5224C>T	P
5159G>A	P/LP
5093_5096delCTAA	NS
4675C>T	P
3640G>T	P
3442delG	P
3257T>G	P
2800C>T	P
1961delA	P
907_910dupGAAT	NS
456_457delCA	P
188T>A	P
135-2A>C	NS

*Iceland founder mutation; **Ashkenazi founder mutation; ***European founder mutation. P/LP: Pathogenic/Likely Patogenic; B: Bening; NS: Not Submitted for *ClinVar* evaluation.
